# Motor Preparation Tracks Decision Boundary Crossing Rather Than Accumulated Evidence in Temporal Decision-Making

**DOI:** 10.1523/JNEUROSCI.1675-24.2025

**Published:** 2025-03-11

**Authors:** Nir Ofir, Ayelet N. Landau

**Affiliations:** ^1^Departments of Psychology, Hebrew University of Jerusalem, Jerusalem 9190501, Israel; ^2^Cognitive and Brain Sciences, Hebrew University of Jerusalem, Jerusalem 9190501, Israel; ^3^Edmond and Lily Safra Center for Brain Sciences, Hebrew University of Jerusalem, Jerusalem 9190401, Israel; ^4^Department of Experimental Psychology, University College London, London WC1H 0AP, United Kingdom

**Keywords:** decision-making, EEG, motor preparation, Mu-beta, temporal bisection, time perception

## Abstract

Interval timing, the ability of animals to estimate the passage of time, is thought to involve diverse neural processes rather than a single central “clock” (Paton and Buonomano, 2018). Each of the different processes engaged in interval timing follows a different dynamic path, according to its specific function. For example, attention tracks anticipated events, such as offsets of intervals (Rohenkohl and Nobre, 2011), while motor processes control the timing of the behavioral output (De Lafuente et al., 2024). However, which processes are involved and how they are orchestrated over time to produce a temporal decision remains unknown. Here, we study motor preparation in the temporal bisection task, in which human (female and male) participants categorized intervals as “long” or “short.” In contrast to typical perceptual decisions, where motor plans for all response alternatives are prepared simultaneously (Shadlen and Kiani, 2013), we find that temporal bisection decisions develop sequentially. While preparation for “long” responses was already underway before interval offset, no preparation was found for “short” responses. Furthermore, within intervals categorized as “long,” motor preparation was stronger at interval offset for faster responses. Our findings support the two-stage model of temporal decisions, where “long” decisions are considered during the interval itself, while “short” decisions are only considered after the interval is over. Viewed from a wider perspective, our study offers methods to study the neural mechanisms of temporal decisions, by studying the multiple processes that produce them.

## Significance Statement

Interval timing is thought to rely on multiple neural processes, yet little is known about which processes are involved and how they are organized in time. We recorded the EEG of human participants while they performed a simple temporal decision task and focused on mu-beta activity, a signature of motor preparation. In typical nontemporal perceptual decisions, mu-beta activity reflects the accumulation of evidence. We find that in temporal decision-making, mu-beta reflects the commitment of the decision instead. This distinction stems from the uniqueness of temporal decisions, in which alternatives are considered sequentially rather than simultaneously. Studying temporal decisions as the dynamic orchestration of multiple neural processes offers a new approach to study the neural mechanisms underlying the perception of time.

## Introduction

Tracking the passage of time is a fundamental capability of animals and forms a scaffold upon which behavior is organized. An “internal clock” underlying all timed behaviors has yet to be described, and a growing body of work suggests such a “clock” might not exist (Paton and Buonomano, 2018). However, any single timing behavior involves multiple processes orchestrated over time, spanning from early perceptual stages to the final motor response, and the perception of time can leave measurable traces in each. Yet, little is known about which neural processes are recruited and how they unfold over time (Kononowicz et al., 2018).

Simple temporal decisions, such as categorizing an interval as “short” or “long,” are thought to involve multiple processes, including “clock” processes which estimate the duration of an interval, memory processes holding exemplars of the categories and decision processes (Treisman, 1963; Gibbon et al., 1984). Early models assumed that intervals are first measured until they are over, and the estimated duration is then transformed into a decision (Machado et al., 2009). However, behavior and EEG provide evidence that participants do not wait until the offset of the interval to decide (Tarantino et al., 2010; Lindbergh and Kieffaber, 2013; Balcı and Simen, 2014; Ofir and Landau, 2022). Instead, participants' behavior is consistent with a two-stage decision process (Balcı and Simen, 2014). The model assumes two sequential accumulation-to-bound stages. In the first stage, a noisy accumulator starts with interval onset and runs until either a decision boundary is reached or the interval ends. If the boundary has been reached, the interval is categorized as “long.” If not, a second noisy accumulation process starts, comparing the accumulated value at the end of the first stage to an internal midpoint. While “long” decisions can be made during the interval or after it, “short” decisions can only be made after the interval offset.

Previously, we have reported an EEG potential which builds up from interval offset to the participants' response, similar to the centroparietal positivity (CPP) signature of evidence accumulation (Ofir and Landau, 2022). Specifically, we found that a larger potential was evoked by intervals that were categorized as “short” than by intervals that were categorized as “long.” In addition, the potential was larger in amplitude for shorter intervals, a pattern that was stronger in “short” compared with “long” trials. In terms of the two-stage model, the offset-evoked potential reflected the distance from the “long” decision boundary at interval offset (Ofir and Landau, 2022), corresponding to the second stage. In the present study, we turn our attention to the first stage of the model. This requires a neural signal that can track “short” and “long” responses separately. The CPP, which reflects general evidence strength, is unsuitable (O’Connell and Kelly, 2021). Previous studies have also found the CPP does not reflect elapsed duration, which is the evidence in temporal decisions (McCone et al., 2024). On the other hand, effector-selective signals of motor preparation, such as mu-beta and the lateralized readiness potential, can shed light on this part of the process.

Mu-beta (8–30 Hz) amplitude is a robust signature of preparing and executing a motor command (Pfurtscheller and Lopes Da Silva, 1999). Specifically, mu-beta amplitude in the hemisphere contralateral to the responding hand decreases gradually toward a fixed level at which movement onsets. While theoretically deciding between alternatives can be implemented in various forms (Bogacz et al., 2006), when different decisions are mapped to different hands, the dynamics of the amplitude resemble that of a race: The amplitude in each hemisphere decreases independently, and the first hemisphere to reach the threshold is the one to respond (O’Connell and Kelly, 2021). Therefore, mu-beta lateralization (i.e., the relative amplitude of mu-beta between the two hemispheres) is taken to reflect differential preparation (and accordingly differential evidence) toward one response alternative versus the other (Donner et al., 2009).

Another effector-selective signature is the lateralized readiness potential (LRP; Smulders and Miller, 2011). The LRP has been found to reflect the formation of decision several hundreds of milliseconds before the response (Afacan-Seref et al., 2018). It has been suggested that the LRP reflects the buildup of temporal decisions as well (Lindbergh and Kieffaber, 2013). However, this result was not statistically significant and deserves additional scrutiny.

## Materials and Methods

This study reports an additional analysis for data that we first analyzed in a previous report (Ofir and Landau, 2022).

### Participants

Forty individuals [23 female; average age, 25 (SD 4.2)] participated in the experiment, corresponding to Experiments 3a and 3b in the original study. Participants were recruited from the university community and were compensated for their time with either money (10 euro per hour) or class credit. All procedures were approved by the institutional review board of ethical conduct. Four participants did not complete one of the tasks (see below, Experimental design and statistical analyses) due to technical reasons, two in each task, resulting in a dataset of 38 participants in each task.

### Stimuli and apparatus

Visual stimuli consisted of a square-wave grating presented in a circular window on a BenQ XL2420Z monitor running on 100 Hz (Experiment 2) using Psychtoolbox in Matlab (MathWorks). The grating had a spatial frequency of three cycles per visual degree, had a diameter of 8° visual degree, and was positioned at the center of the screen. During the experiment, stimuli were presented for different durations (see experimental procedure) at two different levels of contrast, 100 and 50%. Visual contrast is known to affect perceived duration (Matthews et al., 2011); however, adding it as a predictor to our statistical models (see below) did not explain variability in our signals of interest. As such, we collapse across both levels in the rest of the paper. The gratings were presented randomly with a tilt of 45 or 135° and a phase of 0, 90, or 180°.

### Experimental design and statistical analyses

**Figure 1.**
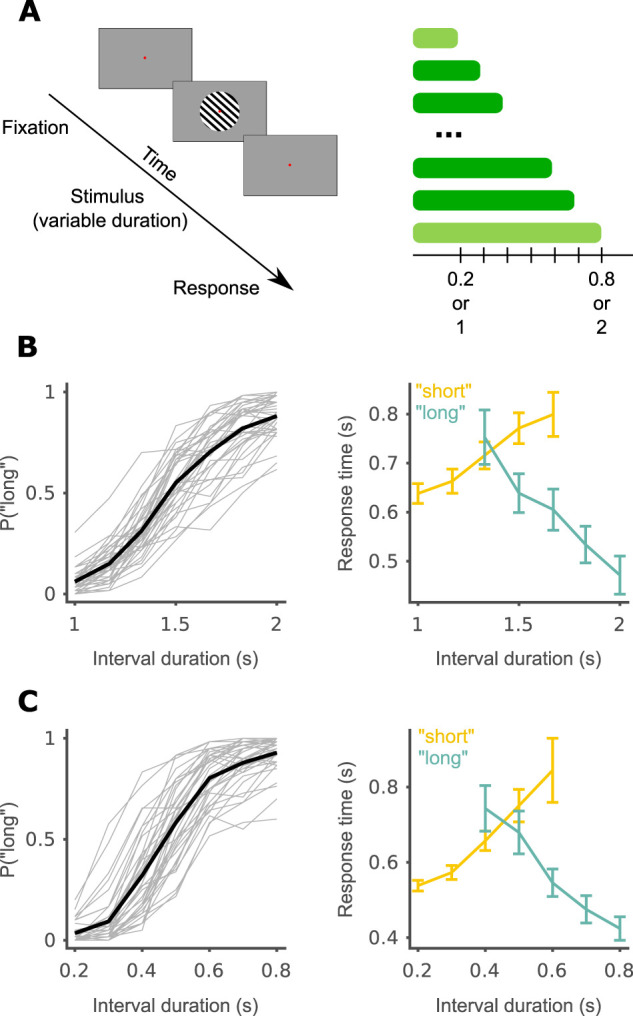
Temporal bisection task. ***A***, Schematic of a single trial (left) and task design (right). Reference intervals are in light green. ***B***, Behavior in the 1–2 s block. Left, Psychometric curve. Single participants in gray and group average in black. Right, Response time as a function of duration and response. Group average and between-participant SEM. Within each response, the two extreme intervals were not plotted as they contain few responses. ***C***, Same as ***B*** for the 0.2–0.8 s block.

The participants performed a visual version of the temporal bisection task (Fig. 1*A*; Kopec and Brody, 2010; Penney and Cheng, 2018). In this task, participants are first trained briefly to identify short and long reference intervals and are then requested to categorize intervals within that range as being more similar to the short or long references. The participants did the task twice, once with 0.2 and 0.8 s as the short and long references, and once with 1 and 2 s as the references, in two separate blocks. The test intervals were [0.2, 0.3, 0.4, 0.5, 0.6, 0.7, 0.8] and [1, 1.17, 1.33, 1.5, 1.67, 1.83, 2] seconds in each block, respectively. The familiarization phase included 12 trials, six per reference duration. The test phase included a total of 420 trials, with 40 trials for each duration. During the entire experiment, participants received feedback for responses to reference durations (the shortest and longest intervals). A self-paced break was given to the participants between the familiarization and test phases and after every 84 trials in the test phase (every ∼10 min). Each interval was presented 12 times within each block of 84 trials in random order. A red fixation point was displayed at the center of the screen (atop the gratings) throughout the entire experiment, excluding breaks. For a trial to start, participants had to fixate within a 1.5° radius of the fixation dot for a continuous second. Participants were asked to respond quickly and accurately after stimulus offset. Participants used a different hand to make “short” and “long” responses, allowing us to look at the dynamics of motor preparation as a window onto the cognitive processes underlying behavior in the task (O’Connell and Kelly, 2021). The response-hand mapping and block order were counterbalanced across participants.

### EEG acquisition

We recorded the EEG of the participants using a g.GAMMAcap (gTec) and a g.HIamp amplifier (gTec). The cap contained 62 active electrodes, positioned over the scalp according to the extended 10–20 system, with the addition of two active earlobe electrodes. We removed electrodes F9 and F10, as they often included strong muscle activity and were far from regions of interest. In addition, for all participants we recorded the horizontal electrooculogram (EOG) using passive electrodes placed at the outer canthi of both eyes and the vertical EOG using electrodes placed above and below the left eye. The EEG was continuously sampled at 512 Hz. We monitored the eye position using an infrared EyeLink camera (SR Research), sampling at 1,000 Hz. The EyeLink signal and the EEG signal were time aligned and stored for offline analysis using a Simulink model (MathWorks).

### EEG preprocessing

The EEG was referenced offline to the average of the earlobes. All offline preprocessing and analyses were done using a combination of FieldTrip (Oostenveld et al., 2011), EEGLAB (Delorme and Makeig, 2004), and custom Matlab code. Bad electrodes were removed by visual inspection. On average, 0.75% of electrodes per participant were removed (max. 6.67%). None of the participants had bad electrodes within the region of interest (C3, C4, CP3, CP4). Slow drifts were removed using a spline-based approach (Ofir and Landau, 2022). Trials were defined from 500 ms before stimulus onset to 500 ms after the participant responded. Artifactual trials were removed using visual inspection guided by the summary statistics, implemented in ft_rejectvisual(). On average, 2.05% (max. 9.05%) and 2.45% (max. 6.43%) of trials were removed per participant in the short references (0.2–0.8 s) and long references (1–2 s) blocks, respectively.

### Extracting signals of interest

In this work, we focus on two motor signals: Mu-beta and LRP. The extraction of the signals closely followed the recent literature (Corbett et al., 2023). First, we applied a surface Laplacian transformation to the EEG (also known as scalp current density) data using the spherical splines approach (Perrin et al., 1989), implemented in ft_scalpcurrentdensity(). Prior to detailed analyses we validated that the signals are measurable in our data (van Ede and Maris, 2016).

**Figure 2.**
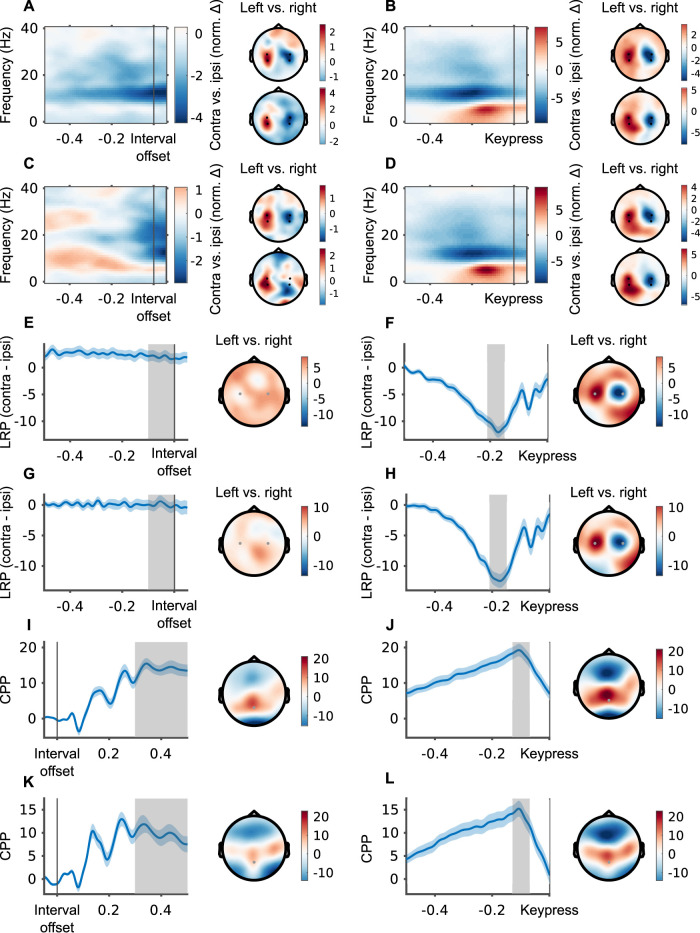
Validation of the mu-beta, LRP and CPP. ***A***, On the left, lateralized time–frequency representation locked to interval offset for the 1–2 s block, using electrodes C3, CP3, C4, and CP4 (as described in the main text). On the right are topographies of the alpha band (8–12 Hz, bottom) and beta band (14–30 Hz, top) amplitudes for left-hand versus right-hand responses. ***B*,** Same as ***A*** but locked to keypress. ***C***, Same as ***A***, for 0.2–0.8 s block. ***D***, Same as ***B***, for 0.2–0.8 s block. ***E***, On the left, LRP locked to interval offset for the 1–2 s block, using electrodes C3 and C4 as described in the main text. On the right is the topography of the difference between left-hand and right-hand responses in the 100 ms before interval offset, marked in gray on the LRP trace. ***F***, Same as ***E*** but locked to keypress. Topography based on the average of 210–150 ms before keypress. ***G***, Same as ***E***, for 0.2–0.8 s block. ***H***, Same as ***F***, for 0.2–0.8 s block. ***I***, On the left, CPP locked to interval offset for the 1–2 s block, using electrode Pz. On the right is the topography of the potential average over all trials 300–500 ms after interval offset, marked in gray on the CPP trace. ***J***, Same as ***I*** but locked to keypress. Topography based on the average of 130–70 ms before keypress. ***K***, Same as ***I***, for 0.2–0.8 s block. ***L***, Same as ***K***, for 0.2–0.8 s block.

To validate that mu-beta is measurable as expected in our data, we computed TFRs (time–frequency representations) and topographies before interval offset and keypress using a short-time Fourier transform, with 300 ms Hann windows, 10 ms steps, and a 1 Hz resolution (by zero padding each 300 ms window to a 1 s window). TFRs were computed for each participant separately for right-hand and left-hand responses and then averaged across participants. To create the topography, we then calculated the normalized difference (i.e., [(left − right)/(left + right)] × 100; Boettcher et al., 2021) within the alpha (8–12 Hz) and beta (14–30 Hz) ranges. To arrive at a single TFR for both hands, we computed the normalized difference of motor channels contralateral and ipsilateral to the response hand for each hand separately [e.g., [(avg(C4, CP4) − avg(C3, CP3))/(avg(C4, CP4) + avg(C3, CP3))] × 100 for right-hand responses] and then averaged across hands. In both blocks, of the 1–2 s intervals and the 0.2–0.8 s intervals, lateralization in the 8–30 Hz band peaked ∼200 ms before keypress, at the expected channels (Fig. 2*B*,*D*). Importantly, the spectrotemporal and spatial patterns of this lateralization were also evident before interval offset (Fig. *2A*,*C*). The offset-locked TFR in the 0.2–0.8 s block was not as clear, since in this case the intervals are much shorter, and the 0.5 s window extends beyond interval onset for many of the intervals we used. For the following analyses of mu-beta, we focused on its lateralization, defined as the difference between the 8 and 30 Hz amplitude in the channels contralateral to the “short” hand and in the channels contralateral to the “long” hand: that is, avg(C4, CP4) minus avg(C3, CP3) for participants who used their right hand for “long” and avg(C3, CP3) minus avg(C4, CP4) for participants who used their left hand for “long.” This definition means positive values indicate lateralization toward the “long” hand and negative values indicate lateralization toward the “short” hand. Using only the “alpha” (8–12 Hz) or “beta” band (14–30 Hz) yielded similar results (Tables 1, 2). Given the temporal averaging inherent in frequency–domain analysis, we used a window of 320–20 ms before keypress (a 300 ms window centered on 170 ms before keypress) and a window of −150–150 ms around interval offset to measure mu-beta lateralization at the time points of interest. As we did not find significant lateralization in the preinterval baseline period (one-sample Wilcoxon signed-rank test, *p* = 0.180) we baselined the lateralization index.

**Table 1.** LMM results for the 1–2 s block

	Offset			Keypress		
	8–12 Hz	14–30 Hz	8–30 Hz	8–12 Hz	14–30 Hz	8–30 Hz
Intercept	**−0.25** (**0.045)**	−0.02 (0.634)	−0.08 (0.210)	−0.20 (0.124)	**−0.17** (**< 0.001)**	**−0.16** (**0.011)**
Duration	−0.05 (0.673)	0.02 (0.599)	0.004 (0.939)	0.14 (0.129)	0.05 (0.222)	0.08 (0.084)
Response	**0.67** (**<0.001)**	**0.31** (**<0.001)**	**0.37** (**<0.001)**	**1.02** (**<0.001)**	**0.69** (**<0.001)**	**0.68** (**<0.001)**
RT	−0.15 (0.367)	−0.07 (0.360)	−0.11 (0.207)	0.11 (0.434)	−0.04 (0.504)	−0.01 (0.844)
Duration by response	0.19 (0.242)	0.04 (0.559)	0.07 (0.386)	−0.14 (0.321)	−0.02 (0.763)	−0.06 (0.366)
RT by response	**−0.58** (**0.011)**	**−0.28** (**0.004)**	**−0.32** (**0.007)**	**−0.59** (**0.003)**	−0.08 (0.334)	**−0.20** (**0.042)**

Each row contains results for a single model parameter. The table is divided into two parts, one for the offset window (left), and one for the keypress window (right). Each cell contains the estimate of the relevant beta coefficient and in parentheses the *p* value for a significance test against zero. Cells with significant coefficients (*p* < 0.05) are marked in bold.

**Table 2.** LMM results for the 0.2–0.8 s block

	Offset			Keypress		
	8–12 Hz	14–30 Hz	8–30 Hz	8–12 Hz	14–30 Hz	8–30 Hz
Intercept	−0.155 (0.230)	−0.086 (0.127)	−0.108 (0.119)	**−0.262** (**0.046)**	**−0.251** (**<0.001)**	**−0.229** (**<0.001)**
Duration	−0.146 (0.193)	−0.090 (0.058)	**−0.119** (**0.033)**	0.007 (0.945)	−0.036 (0.414)	−0.036 (0.486)
Response	0.089 (0.411)	**0.247** (**<0.001)**	**0.201** (**<0.001)**	**0.868** (**<0.001)**	**0.800** (**<0.001)**	**0.737** (**<0.001)**
RT	0.030 (0.851)	0.100 (0.141)	0.068 (0.395)	0.207 (0.160)	0.050 (0.426)	0.087 (0.236)
Duration by response	**0.682** (**<0.001)**	**0.344** (**<0.001)**	**0.426** (**<0.001)**	**0.322** (**0.029)**	0.080 (0.204)	0.141 (0.054)
RT by response	−0.260 (0.234)	**−0.285** (**0.002)**	**−0.246** (**0.024)**	−0.256 (0.205)	**−0.183** (**0.033)**	**−0.197** (**0.049)**

Each row contains results for a single model parameter. The table is divided into two parts, one for the offset window (left), and one for the keypress window (right). Each cell contains the estimate of the relevant beta coefficient and in parentheses the *p* value for a significance test against zero. Cells with significant coefficients (*p* < 0.05) are marked in bold.

To validate that the LRP is measurable as expected in our data, we computed the average potential leading up to the interval offset and up to the keypress. We compared the resulting ERP between trials in which participants responded using their left hand, to those in which they used their right hand. In both blocks, of the 1–2 s intervals and the 0.2–0.8 s intervals, lateralization started ∼500 ms before the keypress, and peaked ∼200 ms before it, at channels C3 and C4 as expected (Fig. 2*F*,*H*). Conversely, we did not observe time courses or topographies resembling the LRP before interval offset (Fig. 2*E*,*G*). Hence, we will only analyze the keypress-locked LRP further. To maintain a consistent representation with the mu-beta lateralization, with positive LRP reflecting execution of a “long” response, we computed the LRP as the channel contralateral to the “short” hand minus the channel contralateral to the “long” hand (i.e., C4-C3 for participants who used their right hand for “long”).

To integrate our analysis here with the results we reported previously (Ofir and Landau, 2022), we also calculated the post-offset CPP. Following the transformation from potential to current density, the topography of the offset-locked and keypress-locked potentials are maximal over parietal electrodes as expected for CPP (Fig. 2*I–L*), rather than the more frontal distribution we and others reported previously (Bueno and Cravo, 2021; Ofir and Landau, 2022; Silvestrin et al., 2022; but see Bannier et al., 2019 for a parietal distribution). We believe the nontransformed topography is shifted toward frontocentral channels due to overlap with occipital visual responses to the offset of the interval.

### Visualizing the effects of decision and RT

**Figure 3.**
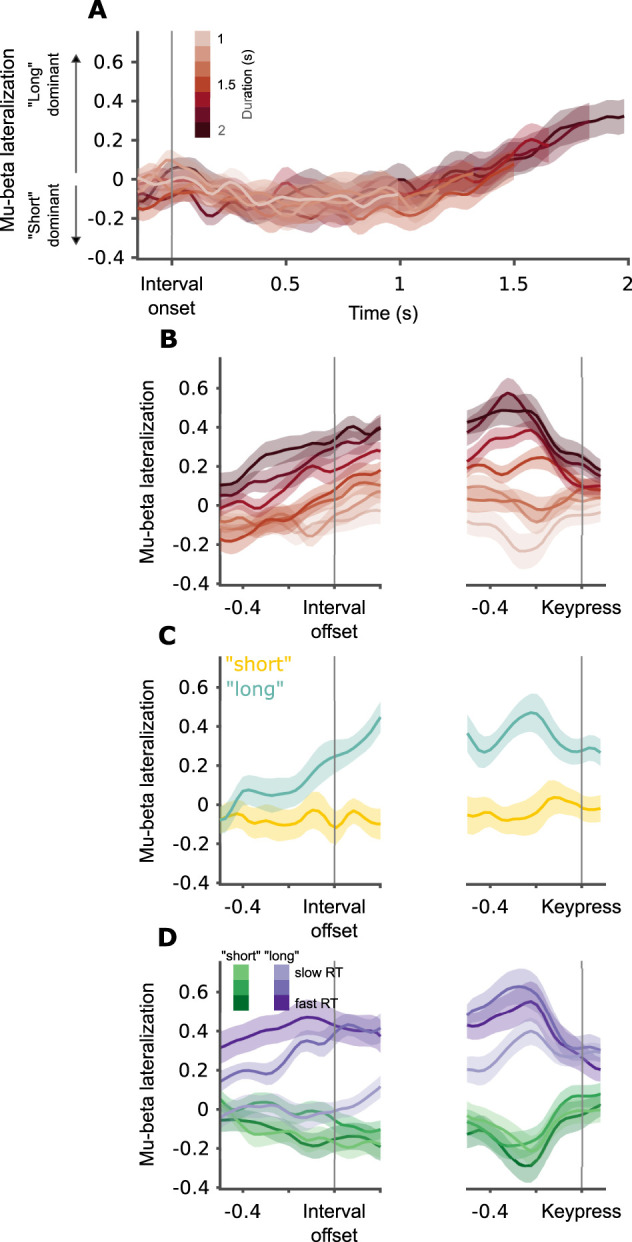
Mu-beta lateralization tracks “long” decisions, 1–2 s block. ***A***, Mu-beta lateralization by interval duration (darker color for longer duration), for the entire interval duration. Grand average and between-participant SEM locked to interval onset. ***B***, Mu-beta lateralization by duration locked to interval offset (left) and keypress (right). Grand average and within-participant SEM. ***C***, Same as ***B***, but for different responses (yellow for “short,” cyan for “long”) at individual bisection points. ***D***, Same as ***B***, but by response (green for “short”, purple for “long”) and RT (darker colors correspond to faster RTs).

The data visualizing the effect of decision (“short” vs “long” trials for the same interval duration; Figs. 3*C*, 5*C*) was calculated by taking the interval duration that was closest to the bisection point for each participant and extracting the signals of interest for those stimuli (Ofir and Landau, 2022). We averaged the signals within participants and then across participants for trials that were categorized as “short” and “long,” separately.

The data visualizing the combined effects of decision and RT (fast vs slow responses, for “short” and “long” trials separately; Figs. 3*D*, 5*D*) was calculated as follows: Within each response (i.e., “short” or “long”), we calculated three equal-size bins of RTs. We then averaged the signals within participants in each of the six pseudoconditions (fast/medium/slow × “short”/“long”) and then across participants.

### Statistical tests

To test for significant lateralization during the first second of the interval in the 1–2 s block, we used a one-sample *t* test against zero, with a false discovery rate correction for multiple comparisons (FDR; Genovese et al., 2002).

To explore the factors affecting mu-beta lateralization, at interval offset and keypress separately for each block, we used linear mixed models implemented in Matlab's fitlme(). All models contained decision (categorical, “short” or “long” with “short” as the reference level), interval duration (continuous, scaled to vary between −1 and 1, with 0 the mean of range: 1.5 for the 1–2 s intervals, 0.5 for the 0.2–0.8 s intervals), and RT (centered within each participant and response). We included random intercepts in all models. The model formula is the following:

$$\begin{array}{r} \mathrm{EEG}=\beta_{0}+\beta_{1}\cdot \mathrm{duration}\;+\;\beta_{2}\cdot \mathrm{decisio}n_{\mathrm{long}}\;+\;\beta_{3}\cdot \mathrm{RT}\\ +\;\beta_{4}\cdot \mathrm{duration}\cdot \mathrm{decisio}n_{\mathrm{long}}\;+\;\beta_{5}\cdot \mathrm{RT}\cdot \mathrm{decisio}n_{\mathrm{long}}. \end{array}$$

The model parameters, then, have the following interpretation:

1.
$\beta_{0}$—lateralization associated with a “short” response at the mean duration and RT.
2.
$\beta_{1}$—the effect of increasing interval duration from the mean to the longest for “short” responses.
3.
$\beta_{2}$—the difference “long”–“short” decisions at the mean duration and RT.
4.
$\beta_{3}$—the effect of increasing RT by 1 s for “short” responses.
5.
$\beta_{4}$—the difference of increasing interval duration from the mean to the longest for “long” responses compared with the effect for “short” responses.
6.
$\beta_{5}$—the difference of increasing RT by 1 s for “long” responses compared with the effect for “short” responses.

## Results

### Mu-beta lateralization tracks “long” but not “short” responses during the interval

We start by describing the dynamics of mu-beta lateralization in the 1–2 s block. From interval onset and up to one second into the interval, mu-beta lateralization remained close to zero (no *p*_corrected _< 0.05; Fig. 3*A*). After 1 s has elapsed from interval onset, a gradually increasing dominance of preparation to a “long” response is apparent. This increase can be visualized succinctly by aligning the traces to interval offset (Fig. 3*B*).

We next explore in more detail the factors underlying mu-beta lateralization in our task. As interval duration is strongly correlated with behavior in this task (Fig. 1*B*,*C*), we used a mixed linear model to identify which experimental and behavioral variables explain mu-beta lateralization at interval offset: interval duration, participant's response (“long”, “short”), and response time (RT), as well as the interaction terms of duration by response and RT by response (full statistical results are presented in Table 1). We found that, once variability related to the behavioral output is considered, interval duration does not explain additional significant variability (Fig. 3*B*; duration predictor, *β* = −0.08, *p* = 0.210; duration by response interaction, *β* = 0.07, *p* = 0.386). Behavioral output, on the other hand, displayed a rich pattern of correlations with mu-beta lateralization. First, we found significant lateralization toward the “long” for intervals categorized as “long,” but there was no significant lateralization for intervals categorized as “short” (response predictor, *β* = 0.37, *p* < 0.001; intercept, *β* = −0.08, *p* = 0.210). This effect is most clearly seen in trials with interval durations that are closest to individual bisection points (the interval for which participants respond “short” on approximately half of the trials). For a fixed duration, clear lateralization is seen before offset for trials with “long” responses. In contrast, for the same fixed intervals, no lateralization is found before interval offset for trials with “short” responses (Fig. 3*C*). Beyond the strong effect of response, response time also predicted lateralization at interval offset. Importantly, the effect of response time was different for “short” and “long” trials. While for “short” trials we did not find a significant association between RTs and lateralization (RT predictor, *β* = −0.11, *p* = 0.207), for “long” trials faster RTs were associated with stronger lateralization at offset (RT by response interaction, *β* = −0.32, *p* = 0.007; Fig. 3*D*).

We next tested whether duration, participants' response, and response time predict the level of lateralization prior to the keypress, using the same linear mixed model. The pattern at keypress differs somewhat from what we found at interval offset. As would be expected from a motor signal, we found significant lateralization for both “short” and “long” responses (intercept, *β* = −0.16, *p* = 0.011; response predictor, *β* = 0.68, *p* < 0.001; Fig. 3*C*). Still, lateralization for “long” responses was larger in absolute terms when compared with “short” responses (paired samples Wilcoxon signed-rank test, *p* = 0.002). Lateralization was stronger for faster “long” responses, but there was no significant association between RTs and lateralization for “short” responses (RT predictor, *β* = −0.01, *p* = 0.844; RT by response interaction, *β* = −0.20, *p* = 0.042; Fig. 3*D*). As at interval offset, interval duration did not significantly predict lateralization (duration predictor, *β* = 0.08, *p* = 0.084; duration by response interaction, *β* = −0.06, *p* = 0.366; Fig. 3*B*).

### Mu-beta lateralization reflects “long” decision boundary crossing

To summarize, mu-beta lateralization during the timed interval presents two important features. First, it starts neutral and remains so during the first second of the timed interval, which corresponds to the shortest interval in the set. From that point and toward the interval's offset, lateralization gradually increases toward “long.” Second, the level of lateralization at interval offset is associated with the RT of “long” responses. This pattern suggests that mu-beta lateralization reflects the commitment of decisions, rather than directly tracking accumulated evidence as has been suggested in nontemporal decision-making (Steinemann et al., 2018).

**Figure 4.**
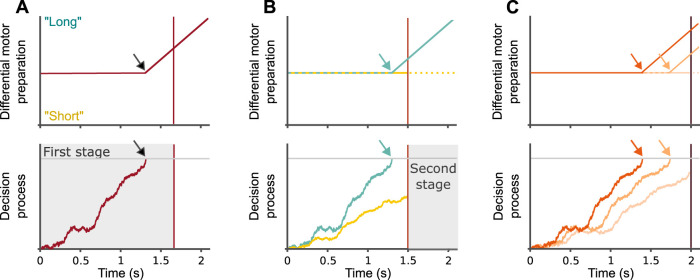
The two-stage model of temporal bisection. ***A***, Model description through an example trial, for an interval lasting 1.67 s. Interval offset is marked by the vertical red line in the bottom and top panels. The decision process is presented in the bottom panel and includes a horizontal gray line signifying the decision boundary. For this trial, the accumulator reaches the boundary at 1.25 s after interval onset, and the interval is categorized as “long.” Motor preparation, in the top panel, is at zero until the boundary is reached, and then a “long” response is prepared. The gray arrows mark the moment in which the accumulator reaches the decision boundary (in the bottom panel) and the moment in which motor preparation begins (in the top panel). In this example, the decision is made in the first stage of the model. ***B***, Model prediction for “short” versus “long” decisions, depicted in yellow and turquoise respectively, using two trials with an equal interval duration of 1.5 s. The accumulator reached the boundary in one trial (turquoise trace), so it is categorized as “long.” In the other trial we assume that the interval is categorized as “short” in the second stage, but we are agnostic about the dynamics of motor preparation in that stage. At interval offset, there is motor preparation toward the “long” hand in the “long” trial, but no motor preparation in the “short” trial. ***C***, Model prediction for RT effect in “long” trials. For example, a single interval, 2 s long, is presented three times. In one trial the boundary is reached before 1.5 s, in the second it is reached after 1.5 s and in the third the boundary is not reached at all. We assume that the third trial is categorized as “long” by the second stage. Reaching the boundary earlier means the motor process has more time to develop until interval offset, resulting in faster RT and stronger lateralization at interval offset.

Our results are expected within the framework of the two-stage decision process that was hypothesized to underlie psychophysical performance in temporal bisection (Fig. 4*A*; Balcı and Simen, 2014). In the first stage of this model, a noisy accumulator starts with interval onset and runs until either it reaches a decision boundary or the interval ends. If the boundary is reached, the interval is categorized as “long” and preparation for the suitable motor response initiates. If it does not, a second stage starts at interval offset. In this stage, the value of the accumulator at interval offset is compared with an internal representation of a threshold between the reference durations. This second stage has two bounds, one for each response (“short” and “long”), and is thought to reflect resampling from memory, as the evidence itself (i.e., interval duration) is no longer directly available (Shadlen and Shohamy, 2016; van Ede and Nobre, 2024).

The basic, and distinguishing, assumption of the two-stage model compared with other models of this task (Gibbon, 1981; Machado et al., 2009; Kopec and Brody, 2010) is that an interval can be categorized as “long” before it ends (i.e., as soon as the decision boundary of the 1st stage is reached) but can only be categorized as “short” after it ends. This explains why “long” responses, but not “short” responses, are associated with significant lateralization at interval offset (Fig. 4*B*). Furthermore, due to the inherent variability of the accumulator during the first stage, there will be trial-to-trial variability in the time it takes to reach the boundary. In trials in which the accumulator rises quickly and reaches the boundary early, motor preparation can start earlier. This will translate into stronger lateralization at interval offset and faster responses. In other trials, in which the boundary will be reached later but still before interval offset, this will translate into weaker lateralization at interval offset and slower RTs. Finally, in some trials, the accumulator will not reach the boundary at all, but the interval will still be categorized as “long” by the second stage. In those trials we expect no lateralization at interval offset and the slowest RTs (Fig. 4*C*). This pattern was indeed what we found: strongest lateralization for the faster “long” responses and essentially no lateralization for the slowest “long” responses (Fig. 3*D*).

### Mu-beta dynamics scale with temporal context

If the mu-beta pattern we find indeed reflects decision boundary crossing, it should depend on when the boundary is crossed. The boundary crossing moment can be experimentally manipulated by changing the temporal context. In the bounded accumulation model of temporal bisection, adapting to different temporal contexts is done by changing the drift rate, while the decision boundary is kept fixed (Balcı and Simen, 2016).

**Figure 5.**
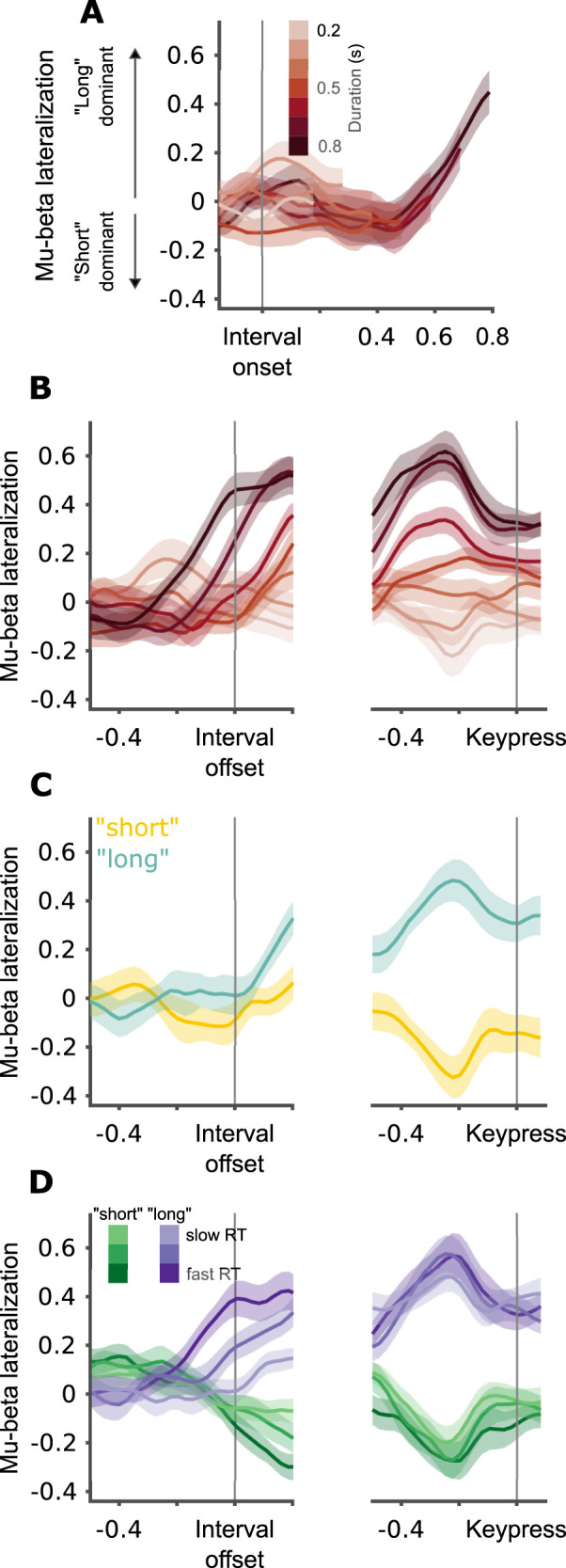
Mu-beta lateralization tracks “long” decisions, 0.2–0.8 s block. ***A***, Mu-beta lateralization by interval duration (darker color for longer duration), for the entire interval duration. Grand average and between-participant SEM locked to interval onset. ***B***, Mu-beta lateralization by duration locked to interval offset (left) and keypress (right). Grand average and within-participant SEM. ***C***, Same as ***B***, but for different responses (yellow for “short,” cyan for “long”) at individual bisection points. ***D***, Same as ***B***, but by response (green for “short,” purple for “long”) and RT (darker colors correspond to faster RTs).

To explore adaptation to temporal context, we turned to the second block in the experiment. In this block the same participants performed the same temporal bisection task on intervals lasting between 200 and 800 ms. The patterns that emerge match those we found using longer intervals but scaled in time to fit the much shorter durations (Fig. 5*A*). We used the same statistical model on the data of this block, and the results generally replicate those we found in the 1–2 s block (full statistical results are presented in Table 2). At interval offset, “long” but not “short” responses were preceded by significant lateralization (intercept, *β* = −0.11, *p* = 0.119; response predictor, *β* = 0.20, *p* < 0.001; Fig. 5*C*). Lateralization was significantly stronger for faster “long” responses, while we did not find a significant association between RT and lateralization in “short” trials (RT response interaction predictor, *β* = −0.25, *p* = 0.024; RT predictor, *β* = 0.07, *p* = 0.395; Fig. 5*D*). The results of the 0.2–0.8 s block diverge from the 1–2 s block with respect to the effect of duration on lateralization. In the 1–2 s block we did not find a significant effect of interval duration. Here, we found that longer intervals were associated with stronger lateralization toward the “long” hand in “long” trials (duration response interaction, *β* = 0.43, *p* < 0.001; Fig. 5*B*). Counterintuitively, longer intervals were associated with stronger lateralization toward the “short” hand in “short” trials (duration predictor, *β* = −0.12, *p* = 0.033). We suspect this results from the pre-offset interval possibly including activity related to stimulus onset, given the short intervals used in this block.

Before keypress, our statistical analysis provided results very similar to those seen in the 1–2 s block. We found significant lateralization for “long” as well as “short” responses (intercept, *β* = −0.23, *p* < 0.001; response predictor, *β* = 0.74, *p* < 0.001; Fig. 5*C*). Lateralization before keypress was stronger for faster “long” responses, while lateralization was not significantly associated with RT for “short” responses (RT by response interaction, *β* = −0.20, *p* = 0.049; RT predictor, *β* = 0.09, *p* = 0.236; Fig. 5*D*). Interval duration did not significantly predict lateralization (duration predictor, *β* = −0.04, *p* = 0.486; duration by response interaction, *β* = 0.14, *p* = 0.054).

### The LRP reflects the final stages of response execution

**Figure 6.**
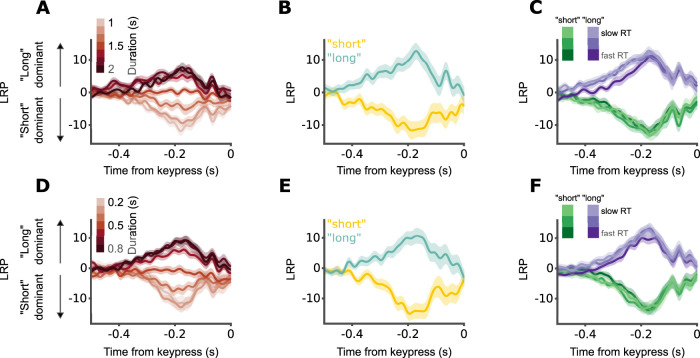
LRP only reflects the final stages of motor execution. ***A***, LRP by interval duration (darker colors for longer durations), for the 1–2 s block. Grand average and within-participant SEM locked to keypress. ***B***, Same as ***A***, but for different responses (yellow for “short,” cyan for “long”) at individual bisection points. ***C***, Same as ***A***, but by response (green for “short,” purple for “long”) and RT (darker colors correspond to faster RTs). ***D–F***, Same as ***A–C***, respectively, but for the 0.2–0.8 s block.

Finally, to complete our analysis of motor signals, we tested whether the LRP amplitude before keypress reflects the interval's duration, the decision of the participant or the RT using the same LMM. The results were the same for both 1–2 s block and 0.2–0.8 s block. We found significant lateralization for “long” as well as “short” responses (1–2 s block: intercept, *β* = −1.630, *p* < 0.001; response, *β* = 2.990, *p* < 0.001; 0.2–0.8 s block: intercept, *β* = −1.990, *p* < 0.001; response, *β* = 3.166, *p* < 0.001; Fig. 6*B*,*E*). Interval duration did not predict LRP amplitude for either “short” or “long” responses (1–2 s block: duration, *β* = −0.062, *p* = 0.646; duration by response interaction, *β* = −0.113, *p* = 0.571; 0.2–0.8 s block: duration, *β* = −0.033, *p* = 0.827; duration by response interaction, *β* = −0.012, *p* = 0.955; Fig. 6*A*,*D*). Similarly, RT for either “short” or “long” responses was not significantly associated with LRP amplitude (1–2 s block: RT, *β* = 0.170, *p* = 0.419; RT by response interaction, *β* = 0.174, *p* = 0.541; 0.2–0.8 s block: RT, *β* = 0.261, *p* = 0.222; RT by response interaction, *β* = −0.457, *p* = 0.117; Fig. 6*C*,*F*). In summary, we find that the LRP only reflects the final stages of motor execution and is not informative about the processes leading up to it.

### CPP and mu-beta reflect different stages of the temporal decision process

**Figure 7.**
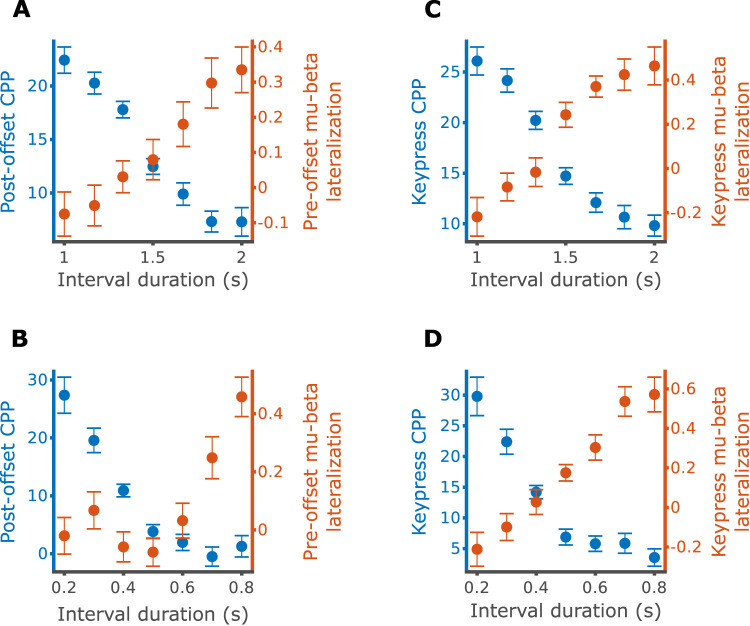
Mu-beta lateralization and CPP display opposite patterns. Mean and within-participant SEM for mu-beta lateralization and CPP amplitude as a function of duration at interval offset, 1–2 s (***A***) and 0.2–0.8 s (***B***) and at keypress, 1–2 s (***C***) and 0.2–0.8 s (***D***).

To integrate the findings here with our previous report, we plotted the CPP amplitude, following interval offset as well as before the keypress, alongside the mu-beta lateralization levels. The two signals display opposite patterns: While the CPP is largest for the shortest duration and decreases as the intervals become longer, mu-beta lateralization is minimal for the shortest duration and becomes more positive as the intervals become longer (Fig. 7). This is so because the CPP reflects distance from decision boundary (i.e., decreasing as internally estimated duration approaches the boundary and saturating for trials in which the boundary has been reached by the offset), while mu-beta lateralization is predicted to be zero until the boundary has been reached. In other words, mu-beta at interval offset lateralizes in trials in which the decision terminates at the first stage, while the CPP arises in trials in which the second stage of the model is active. To relate both signals quantitatively, we used LMMs to predict the CPP amplitude using mu-beta lateralization, after regressing out the effects of interval duration, response, and RT. We found that mu-beta lateralization at interval offset predicted postinterval CPP amplitude significantly only in the 0.2–0.8 s block, where larger lateralization toward the “long” hand associated with smaller CPP amplitude (0.2–0.8 s block: mu-beta, *β* = −0.388, *p* = 0.016; mu-beta by response interaction, *β* = 0.284, *p* = 0.207; 1–2 s block: mu-beta, *β* = −0.065, *p* = 0.652; mu-beta by response interaction, *β* = 0.109, *p* = 0.591). Mu-beta at keypress was not significantly associated with CPP amplitude at keypress in either block (0.2–0.8 s block: mu-beta, *β* = −0.183, *p* = 0.341; mu-beta by response interaction, *β* = 0.300, *p* = 0.263; 1–2 s block: mu-beta, *β* = −0.087, *p* = 0.654; mu-beta by response interaction, *β* = 0.063, *p* = 0.814). In summary, mu-beta lateralization and CPP present only weak correlations once behavior is considered. This supports our proposition that these signatures reflect different stages in temporal decisions.

## Discussion

Timing is a basic cognitive ability which underlies essentially all behavior. We studied how duration is internally represented using the temporal bisection task. This task involves several neural processes, which enables exploring the neural mechanisms of time perception from multiple perspectives. To explore the dynamics of temporal decision formation, we used mu-beta lateralization, which reflects relative motor preparation (Pfurtscheller and Lopes Da Silva, 1999; O’Connell and Kelly, 2021).

From interval onset and up to at least the shortest interval has elapsed, mu-beta was not lateralized toward any choice. From that point, mu-beta gradually lateralized toward the “long” hand. Importantly, this only happened during intervals which were categorized as “long,” regardless of their duration. This pattern suggests mu-beta lateralization reflects the crossing of the “long” decision boundary, rather than the accumulation of evidence toward that boundary.

The neutral starting point of mu-beta lateralization contrasts with a previous report of neuronal recordings from lateral intraparietal neurons in rhesus monkeys performing a similar task (Leon and Shadlen, 2003). In that study, neural activity was found to be biased toward a “short” response initially, with the bias gradually changing into a bias toward “long” over the course of the interval. It is possible that adding a strict response deadline to our design will induce such an initial bias toward “short” in human participants too.

Our finding that motor preparation reflects decision commitment rather than directly tracking the gradual accumulation of evidence seemingly contrasts with multiple studies of nontemporal decision-making (Wyart et al., 2012; Afacan-Seref et al., 2018; Steinemann et al., 2018; Wilming et al., 2020; Balsdon et al., 2023). However, our results are in line with studies using delayed response designs, which find that stronger evidence results in earlier onsets of motor preparation rather than steeper slopes (Twomey et al., 2016; Rogge et al., 2022; see also Balsdon et al., 2021). Temporal bisection shares similarities to several perceptual decision-making designs but is unique. One unique feature of temporal bisection is that decisions in temporal bisections are inherently separated in time: “long” decisions can be committed during the interval, and “short” decision only after it. This is unlike most perceptual decision-making designs, in which all alternative decisions are considered simultaneously (Shadlen and Kiani, 2013). A possible nontemporal analog is a task in which participants need to report whether an interval contained a brief target, which can appear at unpredictable moments or not at all (Landau and Fries, 2012). In this case, participants can decide an interval contained a target as soon as they perceive it but can only judge an interval as not containing a target after the interval elapses. To our knowledge, mu-beta dynamics have not been explored in such designs.

An alternative framing for the bisection task is a “long interval” detection task. This description is especially fitting for the first stage of the computational model and emphasizes the fact that during the interval itself, only buildup of evidence toward a “long” is possible. In this framing, the buildup of mu-beta lateralization toward the “long” response can be thought of as reflecting a nonlinear shift in the starting point of the second stage (Balcı and Simen, 2014). However, such an account would require an additional explanation why mu-beta lateralization was found to increase only for intervals that were categorized as “long.” The two-stage model would predict faster “short” decisions to be associated with starting points closer to the “short” decision boundary, yet we did not find evidence for this.

Shifts in the starting point of decision-making processes can also be caused by prior expectations, which are reflected in a bias in mu-beta lateralization before stimulus onset (de Lange et al., 2013). Future research could study how prior expectations affect temporal decisions using mu-beta lateralization.

We provide here direct neural evidence for the ubiquity of “precommittals”, that is, “long” decisions made before interval offset. This is evident from the fact that “long” decisions display significant lateralization even before the mean interval has elapsed (Fig. 4*C*). We note that modeling the effect of such precommittals is not straightforward. Indeed, the common method of fitting a separate DDM for each duration, or an hierarchical DDM (Balcı and Simen, 2014; Tipples, 2015; Wiener et al., 2018), implicitly assumes that precommittals are negligible, as RTs for precommittals will only reflect motor time, and not a bounded accumulation process.

We found that absolute lateralization before keypress was stronger for “long” compared with “short” responses. A possible explanation is that lateralization before keypress is greater for responses that can be planned in advance (Twomey et al., 2016). This can be tested by delaying responses using a response cue. Delaying responses might also provide a cleaner view on the second stage of decision-making, after interval offset.

Although different EEG signals are used to study distinct cognitive processes, our understanding of the computational role of each signal is preliminary (Cohen, 2017). As we show here, mu-beta and the LRP are clearly distinguished in their temporal extent (Rogge et al., 2022), although both are typically described as related to motor planning. We believe studying the same neural signatures using a wide battery of behavioral tasks will be useful in teasing apart the roles played by each.

Despite being a commonly studied signature, the exact role of the mu-beta band is unknown. It is debated whether and how the mu rhythm, with its strong harmonic in the same band as the beta rhythm, and the beta rhythm can be noninvasively distinguished (Rodriguez-Larios and Haegens, 2023; Schaworonkow, 2023; but see Cheyne, 2013). Studies rarely report both “alpha” (8–12 Hz) and “beta” (13–30 Hz) bands separately, but when they do, activity in both bands is highly similar (Boettcher et al., 2021; Rogge et al., 2022). This similarity suggests that amplitude changes in the beta band in the type of decision-making tasks described here are a result of dynamics of the mu rhythm and not a separate contribution of beta rhythms. Understanding which cognitive and neural processes are related to mu versus beta rhythms is a critical step in ultimately elucidating their computational roles.

Our data highlights the fact that temporal decision-making is dynamic (Balcı and Simen, 2024). Beyond explaining an additional source of RT variability in temporal bisection, we believe this characteristic is critical when examining neural activity in timing tasks. Considering timing behavior as resulting from an orchestration of multiple dynamical processes is a fruitful framework for exploring its neural mechanisms and is in line with current views of other cognitive capabilities (e.g., selective attention; Nobre and Van Ede, 2023).

Integrating this report with our previous study (Ofir and Landau, 2022) paints a fuller picture of temporal decision-making. While mu-beta lateralization reflects the committal of “long” decisions during the timed interval, the CPP reflects the second stage of the decision process starting after interval offset and only if a decision has not been made yet. This second process hypothetically represents sampling from memory, as the interval is already over and no additional evidence is present in the environment (van Ede and Nobre, 2024). The next clearest goal in our view is finding an online signature of elapsed duration, a process that presumably starts at interval onset and ends when mu-beta lateralization begins.
